# National Active Case-Finding Program for Tuberculosis in Prisons, Peru, 2024

**DOI:** 10.3201/eid3103.241727

**Published:** 2025-03

**Authors:** Esther Jung, Valentina A. Alarcón, Wilfredo Santos Solís Tupes, Tatiana Avalos-Cruz, Marco Tovar, Erika Abregu, Max Z. Yang, Jason R. Andrews, Moises A. Huaman

**Affiliations:** Stanford University School of Medicine, Stanford, California, USA (E. Jung, M.Z. Yang, J.R. Andrews); Dirección de Prevención y Control de Tuberculosis (DPCTB), Ministerio de Salud, Lima, Peru (V.A. Alarcón, W.S. Solís Tupes, T. Avalos-Cruz, E. Abregu); Instituto de Investigación Nutricional, Lima (M. Tovar); Escuela de Medicina, Universidad Peruana de Ciencias Aplicadas, Lima (M. Tovar); University of Cincinnati College of Medicine, Cincinnati, Ohio, USA (M.A. Huaman)

**Keywords:** Mycobacterium tuberculosis, tuberculosis and other mycobacteria, bacteria, TB, respiratory infections, antimicrobial resistance, mass screening, rifampicin, asymptomatic infections, Peru

## Abstract

During January–September 2024, a national active case-finding program in Peru’s prisons screened >38,000 persons for tuberculosis (TB) using chest radiography with automated interpretation and rapid molecular tests. The program found high percentages of TB, rifampin-resistant TB, and asymptomatic infections, demonstrating the urgent need for systematic screening among incarcerated populations.

Global tuberculosis (TB) incidence declined over the past decade, but incidence in prisons remained high, and incidence increased in Latin America (*1*). Overcrowding, poor ventilation, and diagnostic delays amplify TB transmission, and incidence among incarcerated persons in Latin America is 27 times higher than among the general population (*2*). The rise in TB in Latin America’s prisons has more than offset reductions in the general population, undermining progress toward international goals to end TB (*3*).

To address the disproportionate TB burden among incarcerated persons, the World Health Organization (WHO) recommended active case-finding for TB in prisons (*4*). However, few standardized nationwide screening efforts have been made in prisons in low- and middle-income countries (LMICs). In Peru, TB screening and treatment has primarily relied on symptom-based screening or passive case detection, without a systematic screening program irrespective of symptoms. In 2023, the Peruvian National TB Program (DPCTB) initiated countrywide screening using chest radiography with computer-detection software, clinical evaluation, and rapid molecular diagnostic testing in high TB–burdened prisons. We evaluated the programmatic yield of that initiative and assessed how each screening component contributed to case identification.

## The Study

**Figure 1:**
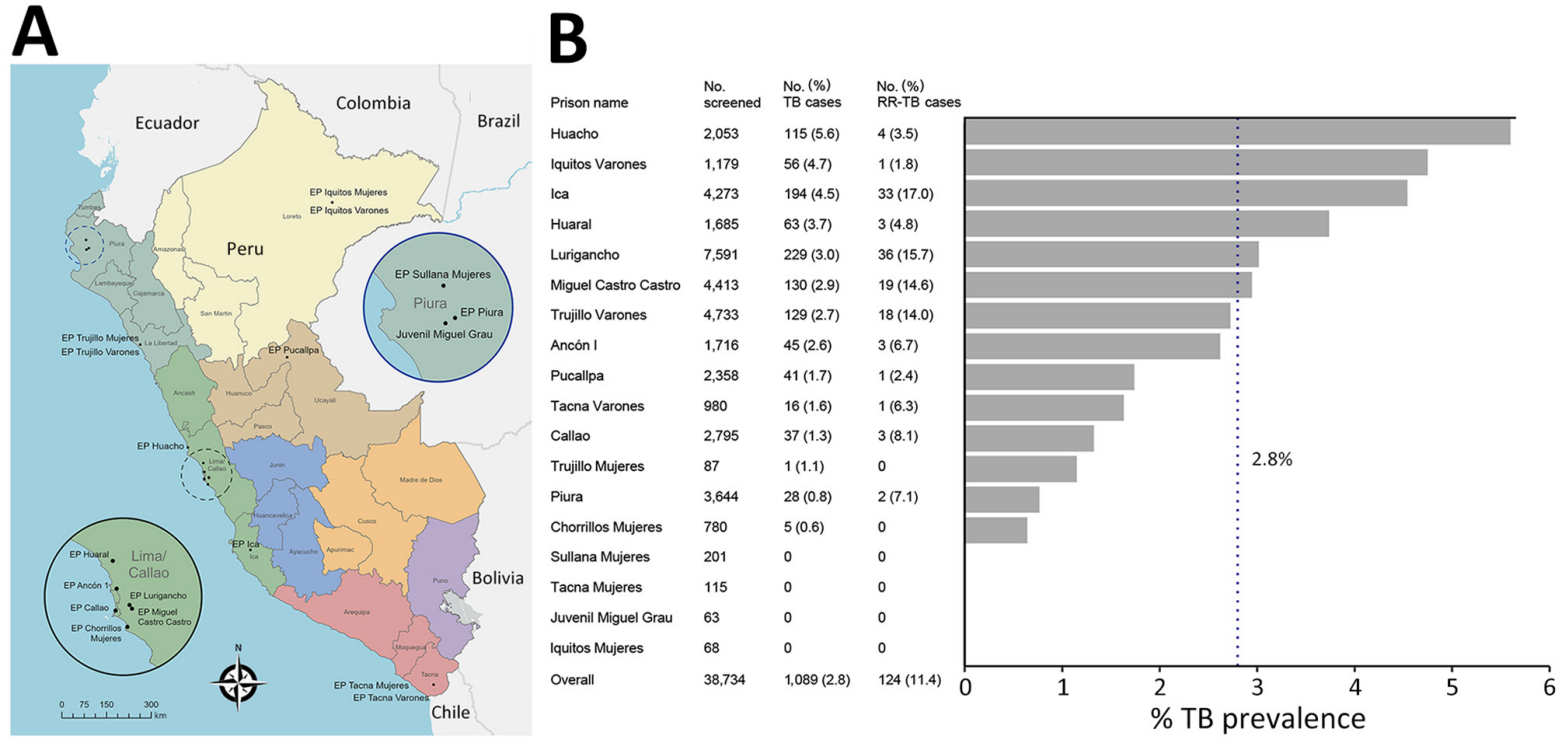
Locations and screening results in a national active case-finding program for TB in prisons, Peru, 2024. A) Locations of 18 facilities included in screening procedures; insets magnify Lima/Callao and Piura departments. B) Bar diagram displaying TB and RR-TB prevalence by prison, sorted by descending TB prevalence. RR-TB percentage is of total TB cases. Dotted line shows overall TB prevalence of 2.8% across all prisons. TB cases were defined as any positive or trace result via Xpert MTB/RIF Ultra (Cepheid, https://www.cepheid.com). EP, Establecimiento Penitenciario (penitentiary establishment); Juvenil Miguel Grau, Centro Juvenil de Diagnóstico y Rehabilitación Miguel Grau; Mujeres, women’s prison, RR-TB, rifampin-resistant TB; TB, tuberculosis; Varones, men’s prison.

Peru has a population of 34 million, among whom 96,805 are incarcerated (*5*,*6*). In 2022, national TB incidence was estimated at 153 cases/100,000 person-years (*6*), but incidence in the prison population was 2,746 cases/100,000 person-years (*7*). In September 2023, DPCTB initiated an active case-finding program in 12 male, 1 co-ed, and 5 female prisons chosen for size, TB burden, and accessibility for DPCTB staff (Figure 1).

We analyzed programmatic data from persons >18 years of age not treated for active TB and screened with chest radiography during January–September 2024. Screening teams included a physician, nurse, and radiology technician. Participants were interviewed for demographic, clinical, and symptom information, then screened with portable digital radiographs that were evaluated by Computer-Aided Detection for Tuberculosis (CAD4TB) version 7.0 (Delft Imaging Systems, https://delft.care). CAD4TB scores radiographs on the basis of abnormalities suggestive of TB. All participants were evaluated by a physician.

Participants with CAD4TB scores >40 (considered abnormal) were asked to produce a sputum sample; participants with scores <40 were only asked for a sputum sample if the physician suspected TB on the basis of symptoms or evaluation. Teams performed rapid molecular diagnostic testing on sputum samples by using Xpert MTB/RIF Ultra assay (hereafter Xpert; Cepheid, https://www.cepheid.com). We defined a TB case as any Xpert-positive result or Xpert result indicating trace *Mycobacterium tuberculosis* DNA levels. All persons with confirmed TB, including drug-resistant TB, received free treatment through directly observed therapy in prison clinics and were isolated in dedicated cells.

**Figure 2:**
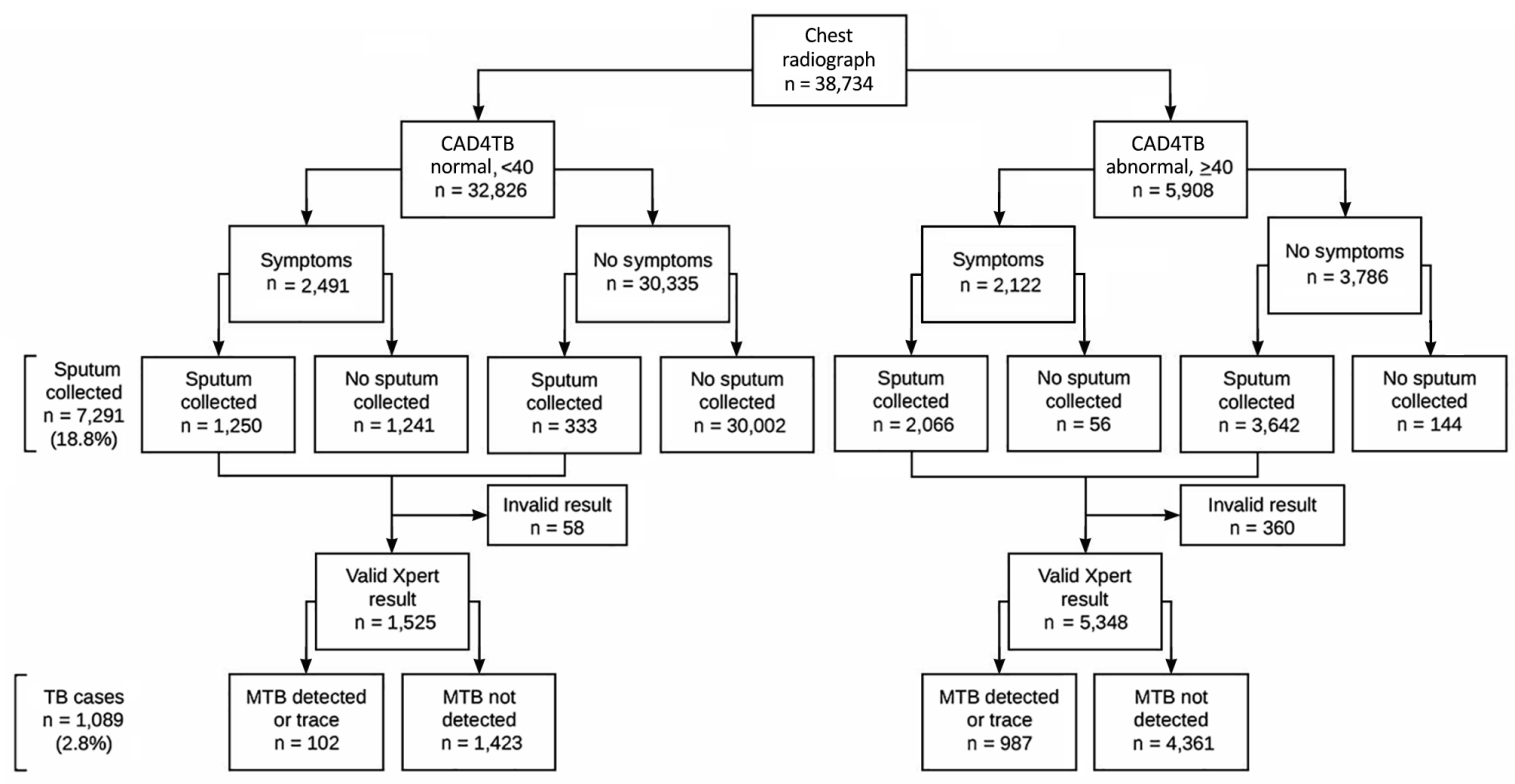
Flowchart for TB screening in a national active case-finding program for tuberculosis in prisons, Peru, 2024. The algorithm shows screening among included participants across 18 study prisons. TB cases were defined as any positive or trace result via Xpert MTB/RIF Ultra (Cepheid, https://www.cepheid.com). CAD4TB, Computer-Aided Detection for Tuberculosis version 7.0 (Delft Imaging Systems, https://delft.care.com); MTB, *Mycobacterium tuberculosis*; TB, tuberculosis; Xpert, Xpert MTB/RIF Ultra (Cepheid, https://www.cepheid.com).

During January–September 2024, DPCTB screened 38,734 eligible participants, representing >80% of the population (48,376 persons) across 18 study prisons (*8*). We collected sputum from 7,291 (18.8%) participants, and 6,873 (94.3%) samples produced valid Xpert results; supply issues at the time of screening prevented Xpert testing for 308 samples (Figure 2). To evaluate demographic and clinical characteristics, we used multivariable logistic regression with fixed effects for prisons to estimate crude odds ratios (ORs) and adjusted ORs (aORs) and 95% CIs, accounting for age, sex, TB history, TB contact, and Peru birth. We calculated sputum positivity and TB case percentages by combinations of symptom screening and CAD4TB results. We used R version 4.4.1 (The R Project for Statistical Computing, https://www.r-project.org) for statistical analyses.

Among participants, 96% were male, 4% were female, 94% were born in Peru, and median age was 35 (IQR 27–43) years. In addition, 16% of participants reported TB history and 40% shared a cell with a known case (Table 1). We diagnosed TB in 1,089 (2.8%) participants. Prevalence ranged from no cases in small prisons (those with <250 persons) to 5.6% in Huacho (population of 2,053). Female prisons had prevalences <1.5%, and 8 of 12 male prisons had prevalences >2%. Among Xpert-positive samples, 11.4% (124/1,089) were rifampin-resistant TB (RR-TB); 4 prisons recorded >10% RR-TB (Figure 1).

**Table 1:** Multivariable logistic regression analysis of demographic characteristics and risk factors for tuberculosis in a national active case-finding program for TB in prisons, Peru, 2024*

Risk factor	No. tested	No. (%) TB confirmed	OR (95% CI)	p value	aOR (95% CI)†	p value
Age group, y						
18–29	11,193	382 (3.4)				
30–44	17,557	486 (2.8)	0.81 (0.70–0.92)	0.002	0.68 (0.59–0.78)	<0.001
45–60	7,708	169 (2.2)	0.64 (0.53–0.77)	<0.001	0.54 (0.45–0.65)	<0.001
>60	2,274	52 (2.3)	0.67 (0.49–0.88)	0.006	0.58 (0.42–0.77)	<0.001
NA	2	0				
Sex						
M	37,227	1,081 (2.9)	5.61 (3.00–12.33)	<0.001	3.78 (1.20–22.94)	0.062
F	1,507	8 (0.5)				
TB history						
Y	6,232	397 (6.4)	3.16 (2.78–3.58)	<0.001	2.75 (2.41–3.13)	<0.001
N	32,502	692 (2.1)				
TB contact						
Y	15,459	556 (3.6)	1.61 (1.43–1.82)	<0.001	1.90 (1.56–2.30)	<0.001
N	23,275	533 (2.3)				
Peru origin						
Y	36,596	1,054 (2.9)	1.80 (1.30–2.57)	0.001	1.67 (1.20–2.41)	0.004
N	2,138	35 (1.6)				

*The program screened 38,734 incarcerated persons during January–September 2024. aOR, adjusted odds ratio; NA, not available; OR, odds ratio; TB, tuberculosis. †aORs include fixed effects for prison and are adjusted for age, sex, TB history, TB contact, and Peru origin.

Among participants, 15.3% (5,908) had abnormal CAD4TB scores, and 11.9% (4,613) reported symptoms in the 2 weeks before participation. Among participants providing sputum samples, Xpert positivity varied substantially by symptom and radiograph status: 6.8% of participants with symptoms but CAD4TB scores <40 accounted for 7.8% of detected cases, 12.2% with abnormal scores but no symptoms accounted for 40.9% of cases, and 26.2% with symptoms and abnormal scores comprised 49.8% of cases (Table 2). Odds of TB were higher among participants with TB history (aOR 2.75, 95% CI 2.41–3.13) and TB contact (aOR 1.90, 95% CI 1.56–2.30) (Table 1). Odds of RR-TB were higher among persons with TB history (aOR 1.96 95% CI 1.32–2.91) (Appendix Table).

**Table 2:** Predictive value of CAD4TB, symptom screening, and sputum testing among participants in a national active case-finding program for TB in prisons, Peru, 2024*

CAD4TB score	Symptoms	No. (%)	Sputum collected	TB confirmed		
				No. confirmed	% TB cases (95% CI)†	% Sputum positivity (95% CI)‡
<40						
	N	30,335 (78.3)	333	17	1.6 (0.9–2.5)	5.1 (3.0–8.1)
	Y	2,491 (6.4)	1,250	85	7.8 (6.3–9.6)	6.8 (5.5–8.3)
≥40						
	N	3,786 (9.8)	3,642	445	40.9 (37.9–43.9)	12.2 (11.2–13.3)
	Y	2,122 (5.5)	2,066	542	49.8 (46.8–52.8)	26.2 (24.4–28.2)
Total		38,734	7,291	1,089		

*CAD4TB, Computer-Aided Detection for Tuberculosis version 7.0 (Delft Imaging Systems, https://delft.care); TB, tuberculosis. †TB case defined as any positive or trace result via Xpert MTB/RIF Ultra (Cepheid, https://www.cepheid). ‡Sputum positivity calculated as percentage of individuals with sputum collected who are confirmed to have TB via Xpert MTB/RIF Ultra (Cepheid).

One limitation of this study is that we did not analyze HIV; however, HIV testing was performed on <5% of participants and prevalence was low (0.01% self-reported; 0 cases among 1,165 screened), consistent with national reports (*9*). Another limitation is that we did not analyze other TB risk factors because of missing or sparse data; however, this implementation study focused on describing the TB burden among the incarcerated population. In addition, the lack of genotype data prevented distinguishing relapse from reinfection, despite previous TB being high among both drug-sensitive and drug-resistant cases. Finally, this study was limited to 18 high-burden prisons, hindering generalizability to other facilities in Peru, and the absence of disease duration data restricted comparisons between study prevalence and prior incidence estimates.

Nonetheless, this study provides TB prevalence estimates in Peru’s carceral system on the basis of molecular testing covering nearly half of the country. Since 2020, Peru has remained among WHO’s 30 countries with the highest burden of drug-resistant TB, and ≈8.3% of new diagnoses annually are drug-resistant (*6*,*10*). Our study detected high (2,800/100,000 persons) TB and RR-TB (11.4%) prevalences. Those findings likely are underestimated because we did not screen persons with previously diagnosed TB or persons on TB treatment, and we only collected sputum from participants with radiographic anomalies, symptoms, or clinical suspicion of TB. In addition, 200 persons with abnormal chest radiographs were not able to provide sputum. Including clinical inference as a criterion would likely have captured additional TB.

Asymptomatic TB is a hidden threat in high-transmission settings like LMIC prisons. Previous estimates suggest that asymptomatic TB represents a considerable proportion of active disease and transmission in general and incarcerated populations (*11*,*12*). In this study, 42.5% of TB cases were asymptomatic, likely an underestimate because of symptom criteria in the screening algorithm, suggesting that symptom-only case-finding would greatly delay or miss active TB infections among incarcerated populations.

## Conclusions

Effective, scalable, active case-finding models are critically needed in LMIC prisons, where nearly half of TB cases go undetected annually (*13*). In Peru, mobile health teams screened >38,000 incarcerated persons in 8 months, covering 40% of the national incarcerated population. That large-scale implementation of active case-finding revealed high prevalences of RR-TB and undiagnosed TB. In addition, 42.5% of persons with Xpert-confirmed TB had no clinical symptoms. Those findings illustrate the importance of systematic TB screening in prisons, particularly for asymptomatic persons.

In summary, our data demonstrated that active case-finding can identify large reservoirs of undiagnosed TB and be performed efficiently at scale in prisons by teams of health professionals. Because TB and RR-TB prevalence is high, intensive screening, including annual or biannual mass screenings, and targeted interventions like TB preventive therapy are essential (*14*,*15*). Such strategies and sustainable financing need to be incorporated into national TB programs to establish, implement, and maintain programs in LMIC prisons, where the TB burden demands urgent attention.

This article was preprinted at https://www.medrxiv.org/content/10.1101/2024.11.08.24317002v1.

Appendix Additional information on national active case-finding program for tuberculosis in prisons, Peru, 2024.
