# Supplementary material for: National Active Case-Finding Program for Tuberculosis in Prisons, Peru, 2024
Source: Emerg Infect Dis. 2025 Mar;31(3):564–8. doi: 10.3201/eid3103.241727 (PMC11878315; doi:10.3201/eid3103.241727)
Supplement: Appendix — Additional information on national active case-finding program for tuberculosis in prisons, Peru, 2024. [file 24-1727-Techapp-s1.pdf]

Article DOI: https://doi.org/10.3201/eid3103.241727

EID cannot ensure accessibility for supplementary materials supplied by authors. Readers who have difficulty accessing supplementary content should contact the authors for assistance.

National Active Case-Finding Program for Tuberculosis in Prisons, Peru, 2024

Appendix

Appendix Table. Multivariable logistic regression analysis of demographic characteristics and risk factors for rifampin-resistant TB among 124 incarcerated persons with confirmed TB*

Risk factor	No. TB confirmed	No. RR-TB (%)	OR (95% CI)	p value	aOR (95% CI)†	p value
Age						
18–29	382	40 (10.5)				
30–44	486	63 (13.0)	1.28 (0.84–1.96)	.256	1.18 (0.76, 1.85)	.462
45–60	169	16 (9.5)	0.89 (0.47–1.62)	.719	0.97 (0.50, 1.82)	.928
>60	52	5 (9.6)	0.91 (0.30–2.23)	.849	1.32 (0.42, 3.45)	.597
Sex						
M	1081	123 (11.4)	0.90 (0.16–16.89)	.922	0.15 (0.01, 3.99)	.192
F	8	1 (12.5)				
TB history						
Y	397	65 (16.4)	2.10 (1.44, 3.06)	<0.001	1.96 (1.32, 2.91)	.001
N	692	59 (8.5)				
TB contact						
Y	556	56 (10.1)	0.76 (0.52, 1.11)	.161	0.59 (0.31, 1.08)	.103
N	533	68 (12.8)				
Peru origin						
Y	1054	122 (11.6)	2.16 (0.65, 13.44)	.294	2.25 (0.65, 14.21)	.279
N	35	2 (5.7)				

*aOR, adjusted odds ratio; CI, confidence interval; OR, odds ratio; RR-TB, rifampin-resistant tuberculosis; TB, tuberculosis.
†aORs include fixed effects for prison, aORs are adjusted for age, sex, TB history, TB contact, and Peru origin.
